# Machine Learning Predicts Femoral and Tibial Implant Size Mismatch for Total Knee Arthroplasty

**DOI:** 10.1016/j.artd.2021.01.006

**Published:** 2021-02-26

**Authors:** Evan M. Polce, Kyle N. Kunze, Katlynn M. Paul, Brett R. Levine

**Affiliations:** a University of Wisconsin School of Medicine and Public Health, Madison, WI, USA; b Department of Orthopaedic Surgery, Hospital for Special Surgery, New York, NY, USA; c Loyola University Chicago, Chicago, IL, USA; d Department of Orthopaedic Surgery, Rush University Medical Center, Chicago, IL, USA

**Keywords:** Templating, Total knee arthroplasty, Predictive modeling, Machine learning, Knee

## Abstract

**Background:**

Despite reasonable accuracy with preoperative templating, the search for an optimal planning tool remains an unsolved dilemma. The purpose of the present study was to apply machine learning (ML) using preoperative demographic variables to predict mismatch between templating and final component size in primary total knee arthroplasty (TKA) cases.

**Methods:**

This was a retrospective case-control study of primary TKA patients between September 2012 and April 2018. The primary outcome was mismatch between the templated and final implanted component sizes extracted from the operative database. The secondary outcome was mismatch categorized as undersized and oversized. Five supervised ML algorithms were trained using 6 demographic features. Prediction accuracies were obtained as a metric of performance for binary mismatch (yes/no) and multilevel (undersized/correct/oversized) classifications.

**Results:**

A total of 1801 patients were included. For binary classification, the best-performing algorithm for predicting femoral and tibial mismatch was the stochastic gradient boosting model (area under the curve: 0.76/0.72, calibration intercepts: 0.05/0.05, calibration slopes: 0.55/0.7, and Brier scores: 0.20/0.21). For multiclass classification, the best-performing algorithms had accuracies of 83.9% and 82.9% for predicting the concordance/mismatch of the femoral and tibial implant, respectively. Model predictions of greater than 51.0% and 47.9% represented high-risk thresholds for femoral and tibial sizing mismatch, respectively.

**Conclusions:**

ML algorithms predicted templating mismatch with good accuracy. External validation is necessary to confirm the performance and reliability of these algorithms. Predicting sizing mismatch is the first step in using ML to aid in the prediction of final TKA component sizes. Further studies to optimize parameters and predictions for the algorithms are ongoing.

## Introduction

Preoperative templating is used by adult reconstruction surgeons to anticipate final implant size for total knee arthroplasty (TKA) and may facilitate enhanced planning capabilities when encountering profound anatomical deformities that necessitate special equipment or custom implants [1,2]. Although templating has become a useful tool for some knee surgeons in preoperative planning because of these benefits, mismatches between planned and final component sizes still occur. In community settings where additional trays may not be readily available, inaccurate preoperative templating may result in longer operative times and an inability to accommodate appropriate implant selection [3,4]. Furthermore, adequate planning may allow for better inventory management and aid the workflow in community hospitals and surgery centers.

With a shift toward value-based health care and the implementation of alternative payment models that impose financial penalties on associated patient morbidity and workflow inefficiency, it is essential to minimize the costs of patient care [5]. The variability in the accuracy of standard preoperative templating, in addition to the costs associated with preoperative imaging and patient-specific instrumentation, [6] poses a challenge to this end and necessitates more efficient and accurate planning methods [5,7]. Previous attempts at demographic-based final implant prediction have demonstrated acceptable accuracy ranging between 71% and 97%, although these models were designed to predict implant ranges of one size under or over the actual implant size [8,9]. Given the range in both the accuracy and variability of implant size predictions in such models, there remains a need for a more accurate and consistent prediction model.

Machine learning is a subset of artificial intelligence that performs tasks that would otherwise necessitate human input [10]. These methods prioritize prediction accuracy through experiential “learning” using real-world data inputs and pattern recognition [11]. These algorithms continually train and assess the accuracy of their predictions, constantly improving their performance with subsequent iterations of analysis. Machine learning has demonstrated considerable potential in the context of value-based health care [12]. By developing advanced prognostic tools for outcome prediction, machine learning overcomes the limitations inherent in traditional regression analyses such as dependence on predefined relationships and collinearity [[13], [14], [15], [16]]. As such, machine learning is particularly poised to be applied to predictive models in orthopedic surgery. A novel concept would be to apply this capability to solve the unmet needs of accurately predicting final implant size in TKA by learning from complex variable relationships in a large data set to optimize predictive performance. The purpose of the present study was to apply machine learning using preoperative demographic variables to predict mismatch between templating and final component size in primary TKA cases. The authors hypothesized that the best performing algorithm would provide moderately increased prediction accuracy of concordance between preoperative templating and final implant sizes based on demographic variables when compared with an experienced adult reconstruction surgeon solely using digital templating.

## Material and methods

### Guidelines

The current analysis was performed according to the Transparent Reporting of a multivariable prediction model for Individual Prognosis or Diagnosis (TRIPOD) guidelines and the Guidelines for Developing and Reporting Machine Learning Models in Biomedical Research [17,18].

### Patient selection

Institutional review board approval at the senior author’s institution was obtained before the commencement of this study. Consecutive patients who underwent primary TKA between 2012 and 2018 with the senior surgeon who had complete preoperative templating data and final implant sizes were included. Exclusion criteria included patients who underwent revision TKA during this time period. Patients underwent TKA at one large academic or 2 community-based hospitals where the senior surgeon (initials blinded for review) operates.

### Templating and implant selection

The primary outcome was mismatch between the final tibial and femoral implant sizes extracted from operative notes and the preoperatively templated tibial and femoral implant sizes. Mismatch was present when the templated tibial or femoral implant size was not the exact same size as the final component implanted intraoperatively. Templating was performed preoperatively by the senior author using OrthoView Preoperative Planning Software (OrthoView, Jacksonville, FL). Preoperative anteroposterior and lateral radiographs with 25-mm calibration markers were uploaded to the software system used for templating. In all cases, the tibia was templated on the lateral radiograph with the goal of maximizing tibial coverage and minimizing tibial overhang in the sagittal plane. Similarly, femoral components were templated on lateral radiographs according to the best-fit anteroposterior dimensions. Previous research has demonstrated that templating is a simple and cost-effective approach in primary TKA [19].

Implants were chosen based off of the senior author’s comfort level with the instrumentation, preoperative planning, and availability at each operating room location. Represented implants in the database included NexGen (Zimmer Biomet, Inc., Warsaw, IN), Vanguard (Zimmer Biomet, Inc., Warsaw, IN), Persona (Zimmer Biomet, Inc., Warsaw, IN), Triathlon (Stryker Corp, Kalamazoo, MI), Evolution (Wright Medical, Inc., Memphis, TN), EMPOWR (DJ Orthopedics, Lewisville, TX), and Attune (DePuy Synthes, Warsaw, IN). Implants were both cruciate-retaining and posterior-stabilized based on the senior author’s preoperative planning and patient-specific factors.

### Covariates and missing data

Height, weight, body mass index, age, gender, and American Society of Anesthesiologists scores were extracted for all patients and used as the combination of features incorporated into algorithm training (Table 1). These variables were selected based off of prior studies investigating prediction of TKA component sizes, which have demonstrated that these demographic variables hold the greatest predictive value [8,9,20]. American Society of Anesthesiologists score was included as it has demonstrated statistical associations with BMI and may therefore have an influence on these parameters that influence component size [21].

**Table 1:** Baseline demographic information of the study population (N = 1801).

Characteristic	Median (IQR) || no. (%)	Rate of missing data (%)
Age, y	63.2 (56.9-69.8)	0
Body mass index, kg/m^2^	33.5 (28.5-40.0)	0.83
Female sex	1225 (68.0)	0
Height, cm	165.1 (160.0-173.7)	0.83
Weight, kg	95.0 (79.4-112.5)	0
ASA classification		0.11
Class I	35 (1.9)	
Class II	924 (51.4)	
Class III	827 (46.0)	
Class IV	13 (0.7)	
Implant type		0
Stryker Triathlon	113 (6.3)	
DePuy Attune	2 (0.1)	
DJ Orthopedics EMPOWR	138 (7.7)	
Biomet Vanguard	64 (3.6)	
Wright Medical Evolution	111 (6.2)	
Zimmer NexGen	1249 (69.4)	
Zimmer Persona	124 (6.9)	
Proportion with femoral component sizing mismatch	538 (29.9)	
Proportion with tibial component sizing mismatch	567 (31.5)	

ASA, American Society of Anesthesiologists; IQR, interquartile range.

Before analysis, the pattern of missing data was diagnosed using Little’s test [22]. The null hypothesis that the data were missing completely at random was rejected (*P* < .001). However, given that no baseline variable exceeded 1% missing data and because the cause of the missing data in this case is unlikely to be related to the missing values, the data were assumed to be missing at random, and multiple imputation with predictive mean matching was performed using the “mice” package in R (R Foundation for Statistical Computing, Vienna, Austria) [23].

### Data preprocessing and model training

An adaptive synthetic sampling approach was implemented during data preprocessing using a heterogenous Euclidean-overlap distance function and a nearest neighbor parameter of 5 to ensure class imbalance was minimized [24]. The primary advantages of using the adaptive synthetic sampling approach compared with other data sampling techniques, such as oversampling and undersampling, are 2-fold: 1) reducing the bias inherent in performing machine learning on imbalanced data sets where often the outcome class of interest is the minority class, and 2) using a dynamic weighted parameter to focus model training on minority class examples that are relatively more difficult to learn. After data-preprocessing, the data set was partitioned randomly using an 80:20 split into a training (n = 1441) and independent testing (hold-out) set (n = 360) for model training and internal validation, respectively. In accordance with previous literature, a minimum of 200 events and 200 nonevents were included before initiation of model training and internal validation [25]. The following 5 machine learning models were developed using 3 iterations of 10-fold cross-validation on the training set: 1) stochastic gradient boosting (SGB), 2) random forest, 3) support vector machine, 4) neural network, and 5) elastic-net penalized logistic regression. Briefly, the training data set is first split into 10 subsets (referred to as “folds”). Each model is trained on 9 of the folds and then tested on the fold that was not used for model training. This process occurs for all 10 of the folds and then is repeated 3 times, which prevents overfitting and enhances the generalizability of the model. Computational differences between these 5 models have been described in detail in previously published literature [[26], [27], [28], [29], [30]].

### Evaluation of model performance

After model development on the training set, the performance of each model was evaluated on the independent testing (hold-out) set of patients. Metrics used to assess model performance, including discrimination, calibration, Brier score, and decision curve analysis, have been described previously [26,31]. Briefly, discrimination was evaluated using receiver operating characteristic (ROC) curve with area under the curve (AUC) analysis [32,33]. In the present study, the AUC represents the probability that the model predicts a greater likelihood of component mismatch to a randomly selected positive case (ie, patient had a component sizing mismatch) than a randomly selected negative case (ie, patient did not have a component sizing mismatch). For reference, an AUC value of 1 corresponds to perfect discrimination, whereas an AUC value of 0.5 indicates that the model classifications were no better than random chance.

While discrimination quantifies how well the model differentiates between those with and without the outcome of interest, calibration corresponds to the accuracy of the model risk estimates. For example, a model that exhibits excellent discrimination between outcome classes (ie, those that do and do not experience the outcome of interest) may simultaneously overestimate the risk of experiencing the outcome for all patients, leading to good discrimination but poor calibration. Two metrics used to assess calibration include the calibration intercept and calibration slope [32,33]. The calibration intercept measures the tendency of the model risk estimates on average to either overestimate or underestimate the observed outcome prevalence. The calibration slope indicates whether the risk estimates were too moderate or too extreme. A calibration intercept of 0 and calibration slope of 1 denote perfect model calibration.

The Brier score constitutes an estimate of overall model performance and is an extension of calibration. The Brier score is calculated by taking the mean of the squared differences between the predicted risk estimates of the model and the corresponding ground truth outcomes [34]. Accordingly, lower Brier scores are indicative of better calibration and overall model performance. The Brier scores for each algorithm were then compared with the null model Brier score, with the null model representing a model where the predicted risk estimate is simply the outcome prevalence in the study population.

Decision curve analysis is used to determine the “net benefit” of prediction models relative to default management strategies, such as treating all or no patients [35]. The net benefit is calculated along a continuum of risk threshold probabilities, which are defined as the minimum likelihood of the outcome (ie, component sizing mismatch) that would justify clinical intervention (ie, reassessment of the patient’s preoperative component template). In contrast to discrimination and calibration, net benefit reflects whether or not the prediction model *should* or *should not* be used in the clinical setting by weighing the consequences of model decisions and potential misclassifications. A further discussion of the utility and interpretation of decision curves can be found in the study by Vickers et al. [35,36].

### Determination of risk estimate classes

A 3-tier class designation was created to risk-stratify patients based on the predicted probability of component mismatch calculated by the model. ROC curve analysis was used to determine the predicted probability threshold that optimally differentiated between those with and without component mismatch using the “cutpointr” package. The “maximize sensitivity” parameter was used to select the optimal threshold, and 1000 bootstrap samples were computed to generate the threshold 95% confidence interval. The risk classes were determined based on the model predicted probabilities for patients in the testing (hold-out) set, defined as the following: 1) low risk, predicted probabilities less than the lower bound of the 95% confidence interval for the optimal threshold; 2) moderate risk, predicted probabilities greater than the “low risk” class upper boundary but less than the third quartile (75th percentile) of the predicted probabilities for the patients without the outcome of interest (ie, no component mismatch); and 3) high risk, predicted probabilities greater than the “moderate risk” class upper boundary.

### Assessment of model fidelity and digital application development

Although there has been a steady increase in the use and adoption of machine learning models in medical research, the “black box” nature of these models limits their interpretability and, consequently, may decrease trust in the predictions. To combat this limitation, Ribeiro et al. [37] introduced local interpretable model-agnostic explanations to explain how machine learning models arrive at individual classification decisions. Briefly, the machine learning model is evaluated through stratifying continuous input variables into a discrete number of bins and using permutations to sample the relationship between the input variables and corresponding model predictions. Owing to the complexity of the machine learning model, an “interpretable” model is then fit to these observations, and model fidelity is assessed locally (ie, how closely the interpretable model reflects the global behavior of the machine learning model in a particular instance). The weighted importance of each input variable is then used to generate plots that provide explanations of model behavior for individual predictions. In the present study, the explanation fit was maximized using the Manhattan distance function and kernel width of 3.0.

An open-access web-application was subsequently created to provide risk predictions and explanations based on individual patient data, freely available at the following link: https://orthopedics.shinyapps.io/TKA_Mismatch/. In its current state, the application merely represents an educational tool for future research, and using this tool is not recommended until external validation in different geographical locations and patient populations is performed. The tool is simply meant to serve as a proof-of-concept demonstration and is not recommended nor appropriate for clinical use at present.

All statistical analyses were performed using the computing software R (R version 1.2.5033, R Foundation for Statistical Computing, Vienna, Austria).

## Results

### Baseline demographics of study population

A total of 1801 patients who underwent TKA were included in the analysis. The study population had a median (interquartile range) age and body mass index of 63.2 (56.9-69.8) years and 33.5 (28.5-40.0) kg/m^2^, respectively, and 68.0% were female. The percentage of patients with femoral and tibial component size mismatch was 29.9% and 31.5%, respectively (Table 1), indicating that surgeon templating was accurate 70.1% and 68.5% of the time for predicting exact component size.

### Algorithm performance for multiclass and binary classification

**Figure 1:**
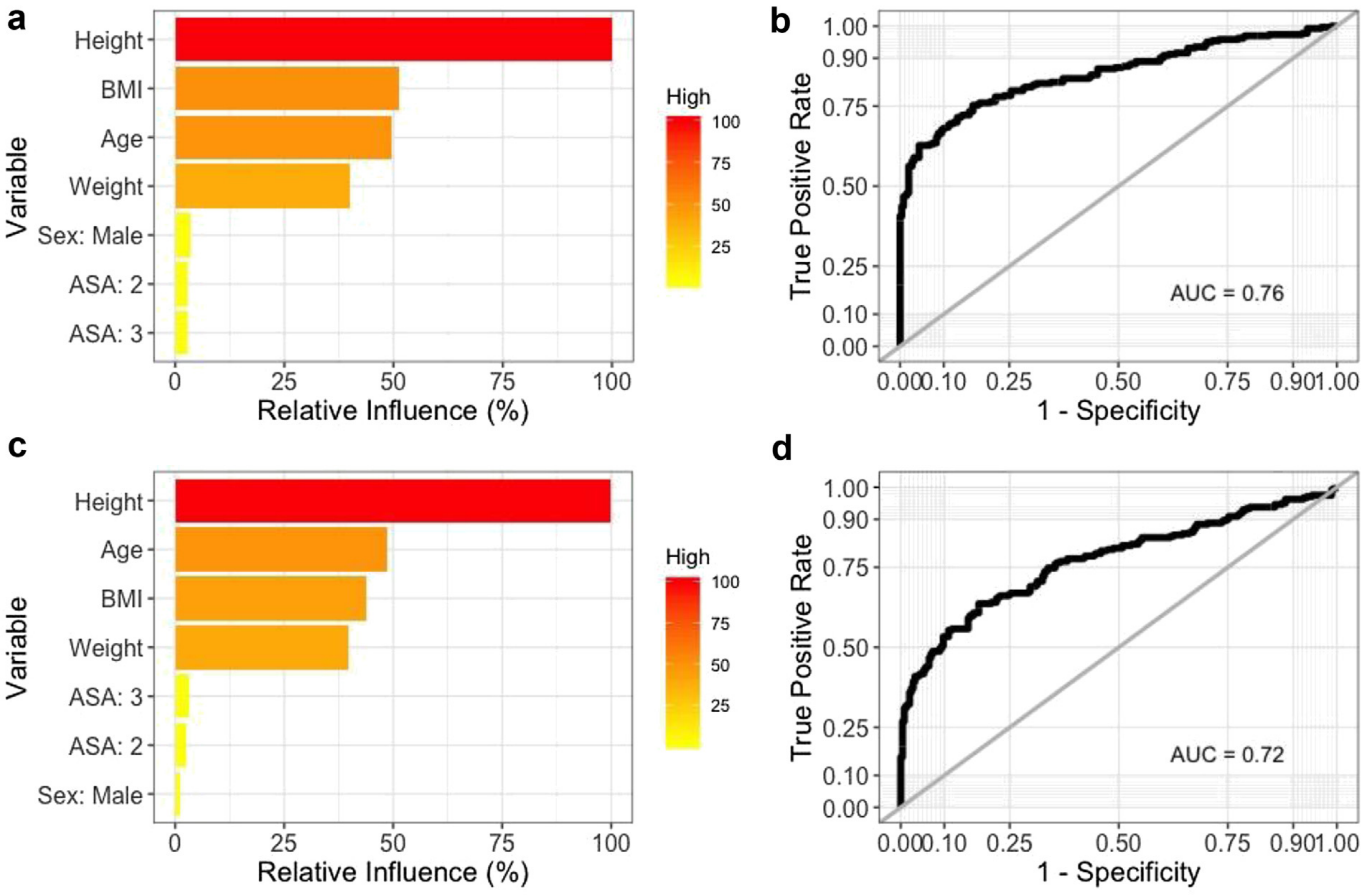
(a, c) Global variable importance plots and (b, d) receiver operating characteristic curves for predicting femoral and tibial component mismatch, respectively, on the independent testing (hold-out) set (N = 360). The predictive importance of each feature is compared relative to other predictor variables included in the model. Red shading indicates variables with greater importance to the model predictions, whereas yellow shading indicates less relative importance.

**Figure 2:**
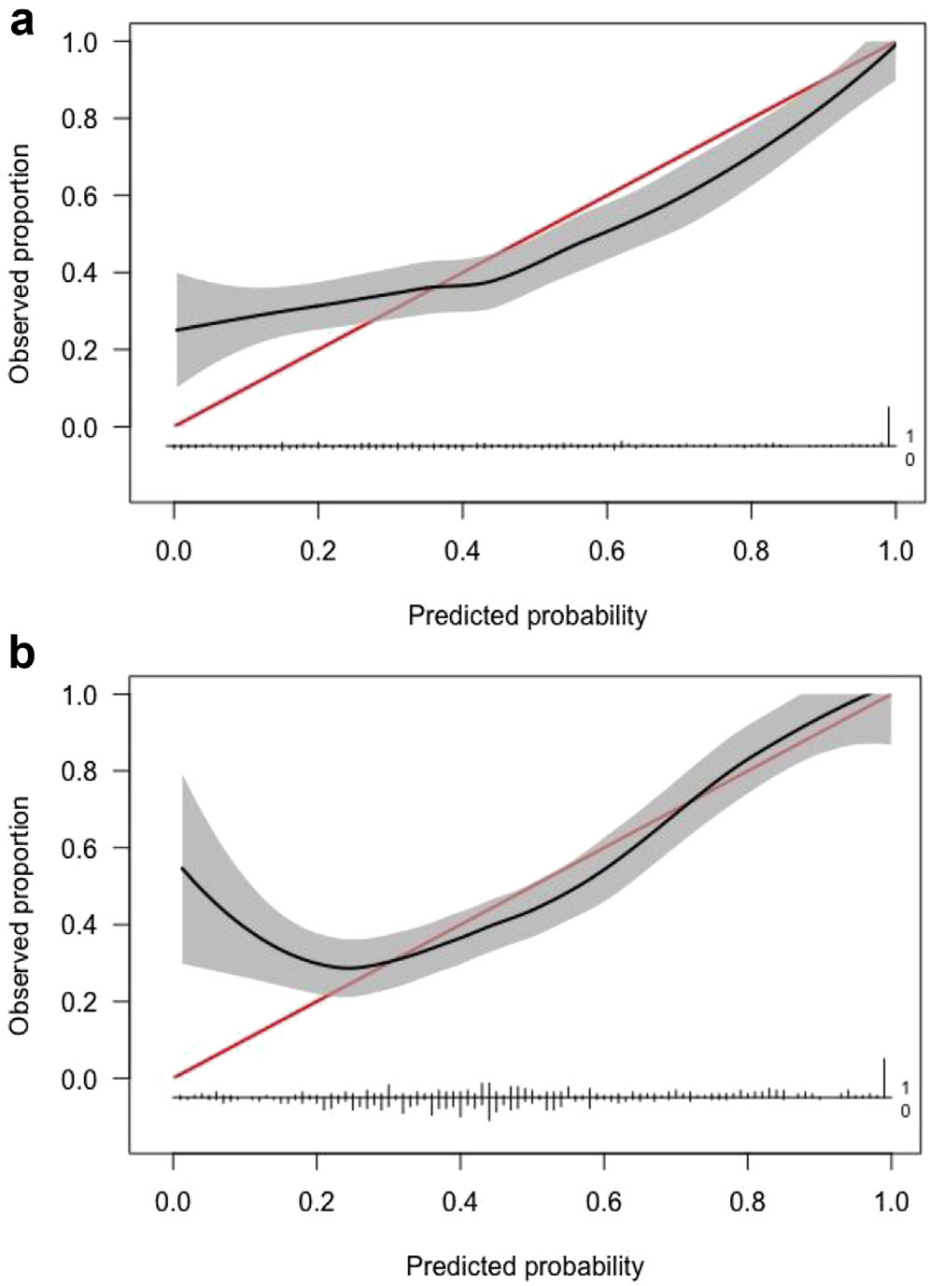
Plot displaying the stochastic gradient boosting (SGB) model calibration for predicting (a) femoral and (b) tibial mismatch when tested on the independent hold-out set not used for model training. The observed probabilities of femoral/tibial component mismatches are shown on the y-axis, with the frequency distribution of the corresponding predicted probabilities made by the SGB model displayed on the x-axis. The 95% confidence interval for the calibration line is represented by the gray shading.

**Figure 3:**
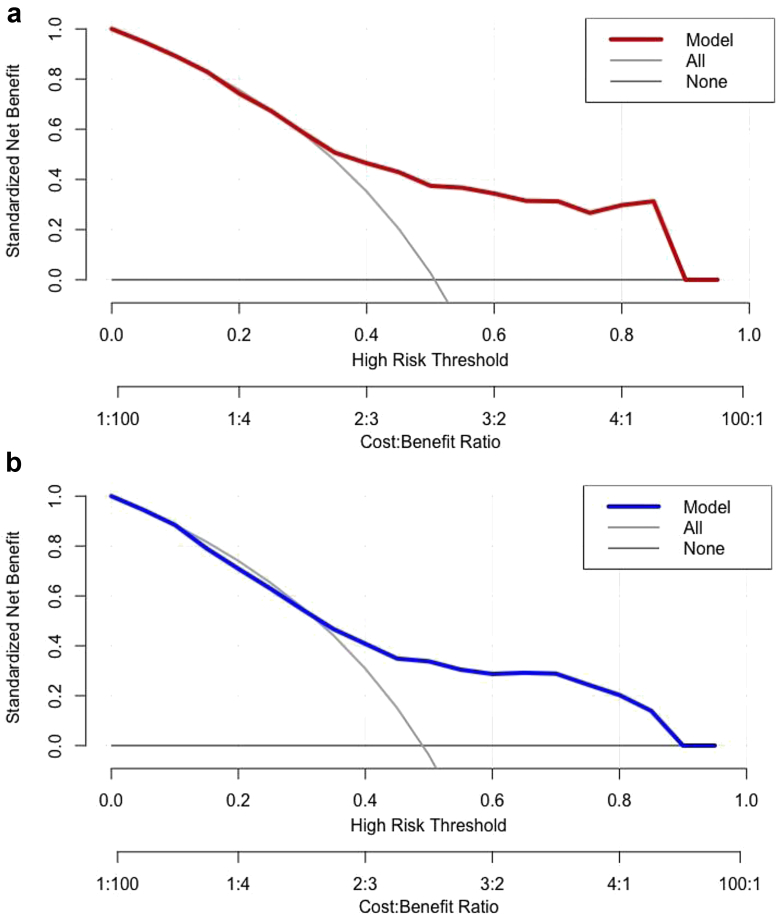
Decision curve analysis plot showing the standardized net benefit of using different management strategies: 1) the stochastic gradient boosting model for predicting (a) femoral and (b) tibial component mismatch; 2) managing all patients as if they had femoral/tibial component mismatch (all), and 3) managing all patients as if they did not have femoral/tibial component mismatch (none). The x-axis demonstrates the standardized net benefit of each management strategy as the weight of false-positive designations (ie, instances where the model predicts a component mismatch incorrectly) increase from low (risk threshold [RT]: 0.0, cost-to-benefit ratio [CTBR]: 1:100) to high (RT: 1.0, CTBR: 100:1). The stochastic gradient boosting model results in greater net benefit than the other management strategies between the risk thresholds of 0.35 to 0.9.

The relative performances of the 5 machine learning models for prediction of binary (yes vs no) femoral/tibial component sizing mismatch are displayed in Table 2, Table 3. A subanalysis to determine the efficacy of multiclass (underfitted vs neutral vs overfitted) femoral/tibial sizing mismatch was also performed (Table S1, Table S2). With respect to binary performance, the best algorithm in terms of discrimination, calibration, and overall performance for predicting femoral and tibial mismatch was the SGB model. Variable importance plots indicated that the factor with the greatest importance for predicting both femoral and tibial component mismatch was height (Fig. 1A and C). The model had AUC values of 0.76 and 0.72 (Fig. 1B and D), calibration intercepts of 0.05 and 0.05, calibration slopes of 0.55 and 0.7 (Fig. 2), and Brier scores of 0.20 and 0.21. The null model Brier score for reference was 0.25, indicating that the algorithm predictions were adequately calibrated. Decision-curve analysis demonstrated that using the SGB models for predicting component mismatch conferred greater net benefit than the alternate management strategies of managing patients as if 1) all had component mismatch and 2) none had component mismatch (Fig. 3). The results of a subanalysis of model accuracy for predicting femoral and tibial component size mismatch stratified by implant type/manufacturer are displayed in Supplemental Table 3.

**Table 2:** Binary classification performance for predicting femoral component sizing mismatch.

Performance metric	Stochastic gradient boosting	Random forest	Support vector machine	Neural network	Elastic-net penalized logistic regression
AUC	0.76 (0.71, 0.80)	0.73 (0.71, 0.79)	0.66 (0.61, 0.71)	0.69 (0.65, 0.74)	0.69 (0.64, 0.73)
Calibration intercept	0.05 (-0.17, 0.27)	0.30 (0.10, 0.51)	0.10 (-0.09, 0.28)	0.04 (-0.15, 0.24)	0.05 (-0.14, 0.24)
Calibration slope	0.55 (0.42, 0.68)	0.79 (0.59, 0.99)	1.14 (0.84, 1.45)	0.95 (0.56, 1.34)	0.98 (0.64, 1.33)
Brier score	0.20 (0.18, 0.22)	0.20 (0.18, 0.21)	0.21 (0.20, 0.22)	0.21 (0.19, 0.22)	0.21 (0.20, 0.22)
Accuracy	0.70 (0.66, 0.74)	0.71 (0.66, 0.75)	0.64 (0.60, 0.68)	0.64 (0.60, 0.68)	0.63 (0.59, 0.67)

AUC, area under the curve.

Null model Brier score = 0.25.

**Table 3:** Binary classification performance for predicting tibial component sizing mismatch.

Performance metric	Stochastic gradient boosting	Random forest	Support vector machine	Neural network	Elastic-net penalized logistic regression
AUC	0.72 (0.67, 0.76)	0.70 (0.66, 0.75)	0.63 (0.58, 0.68)	0.58 (0.53, 0.63)	0.56 (0.51, 0.61)
Calibration intercept	0.05 (-0.15, 0.25)	0.08 (-0.13, 0.28)	-0.03 (-0.21, 0.15)	0.05 (-0.14, 0.23)	0.00 (-0.18, 0.18)
Calibration slope	0.70 (0.50, 0.90)	0.66 (0.46, 0.85)	1.42 (0.89, 1.95)	0.79 (0.27, 1.30)	0.67 (0.25, 1.09)
Brier score	0.21 (0.19, 0.22)	0.22 (0.20, 0.23)	0.24 (0.23, 0.24)	0.24 (0.22, 0.25)	0.24 (0.23, 0.25)
Accuracy	0.67 (0.62, 0.71)	0.66 (0.62, 0.71)	0.63 (0.58, 0.67)	0.59 (0.54, 0.63)	0.55 (0.50, 0.59)

AUC, area under the curve.

Null model Brier score = 0.25.

### Determination of risk-stratified class designations

**Figure 4:**
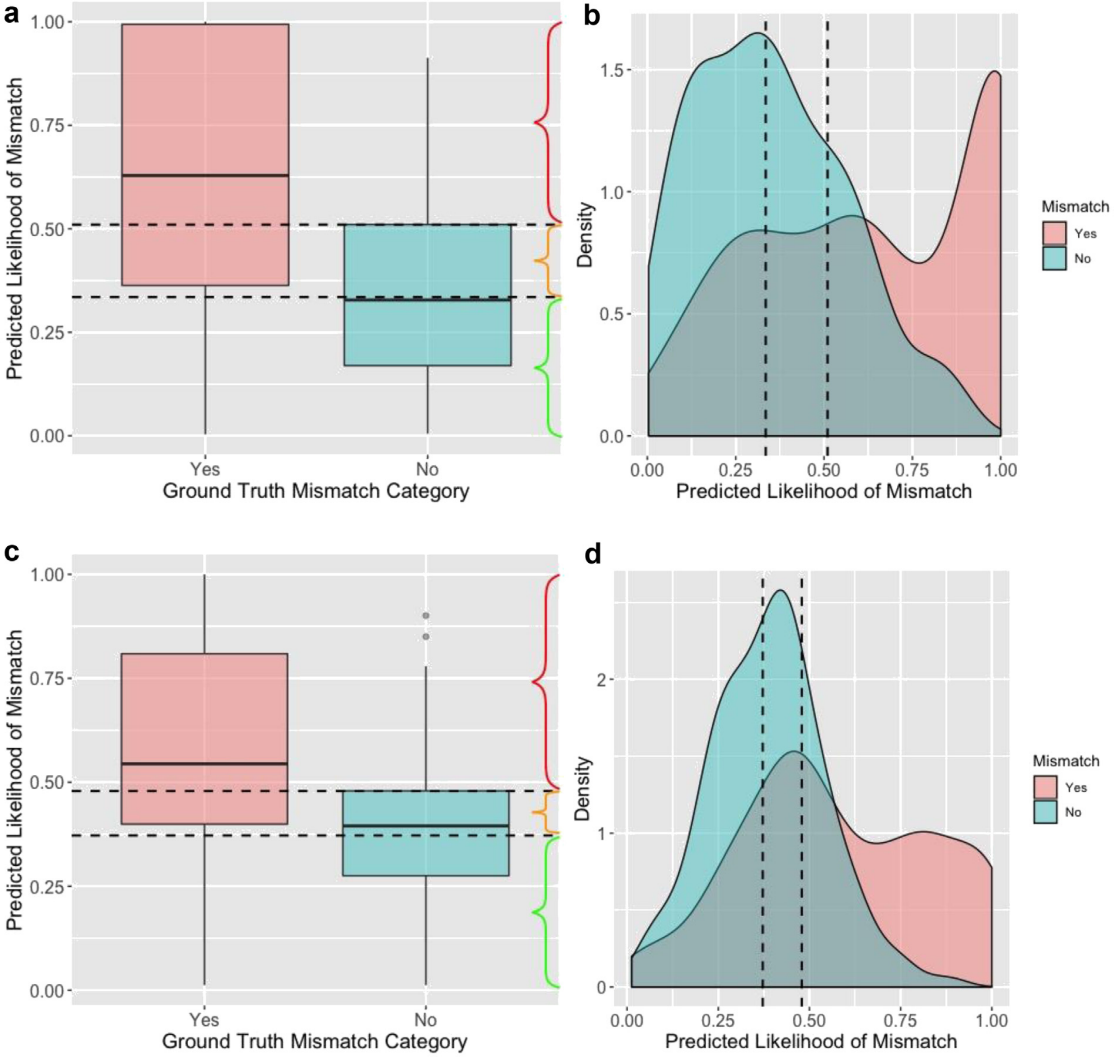
(a, c) Boxplots displaying the predicted probabilities of femoral and tibial mismatch, respectively, made by the stochastic gradient boosting (SGB) model for patients with (yes) and without (no) observed sizing mismatch in the independent testing set not used for model training. (b, d) Density plots showing the probability density function of the predicted probabilities of femoral and tibial mismatch, respectively, by the SGB model for patients with and without observed sizing mismatch. The dashed lines represent the prediction class boundaries. The qualitative class designations based on the SGB predictions are as follows: 1) low risk of component sizing mismatch, green brackets; 2) moderate risk of component sizing mismatch, orange brackets; and 3) high risk of component sizing mismatch, red brackets.

Using the SGB model predictions, qualitative class designations were constructed to provide context to the predicted probabilities of component mismatch (Fig. 4). ROC analysis indicated that the predicted probabilities above which patients are at a greater risk for femoral and tibial component mismatch were 33.5% and 39.4%, with corresponding sensitivities for correctly identifying sizing mismatch of 77.0% and 75.3%, respectively. A 3-tier, qualitative class designation scale was subsequently created to risk-stratify patients into the following categories: 1) low risk of component sizing mismatch, 2) moderate risk of component sizing mismatch, and 3) high risk of component sizing mismatch (Fig. 4A and C; Table 4).

**Table 4:** Risk-stratified classes corresponding to the likelihood of femoral and tibial component sizing mismatch.

Component	Optimal threshold^a^ (%)	Cutoff sensitivity (%)	Low risk	Moderate risk	High risk
Femoral	33.5 (31.3, 36.2)	77.0 (70.9, 83.2)	Less than 31.3%	31.3%-51.0%	Greater than 51.0%
Tibial	39.4 (37.2, 41.1)	75.3 (68.5, 82.0)	Less than 37.2%	37.2%-47.9%	Greater than 47.9%

a The optimal threshold corresponds to the predicted model probability above which patients are at a greater risk for component mismatch.

### Patient-specific risk prediction application

**Figure 5:**
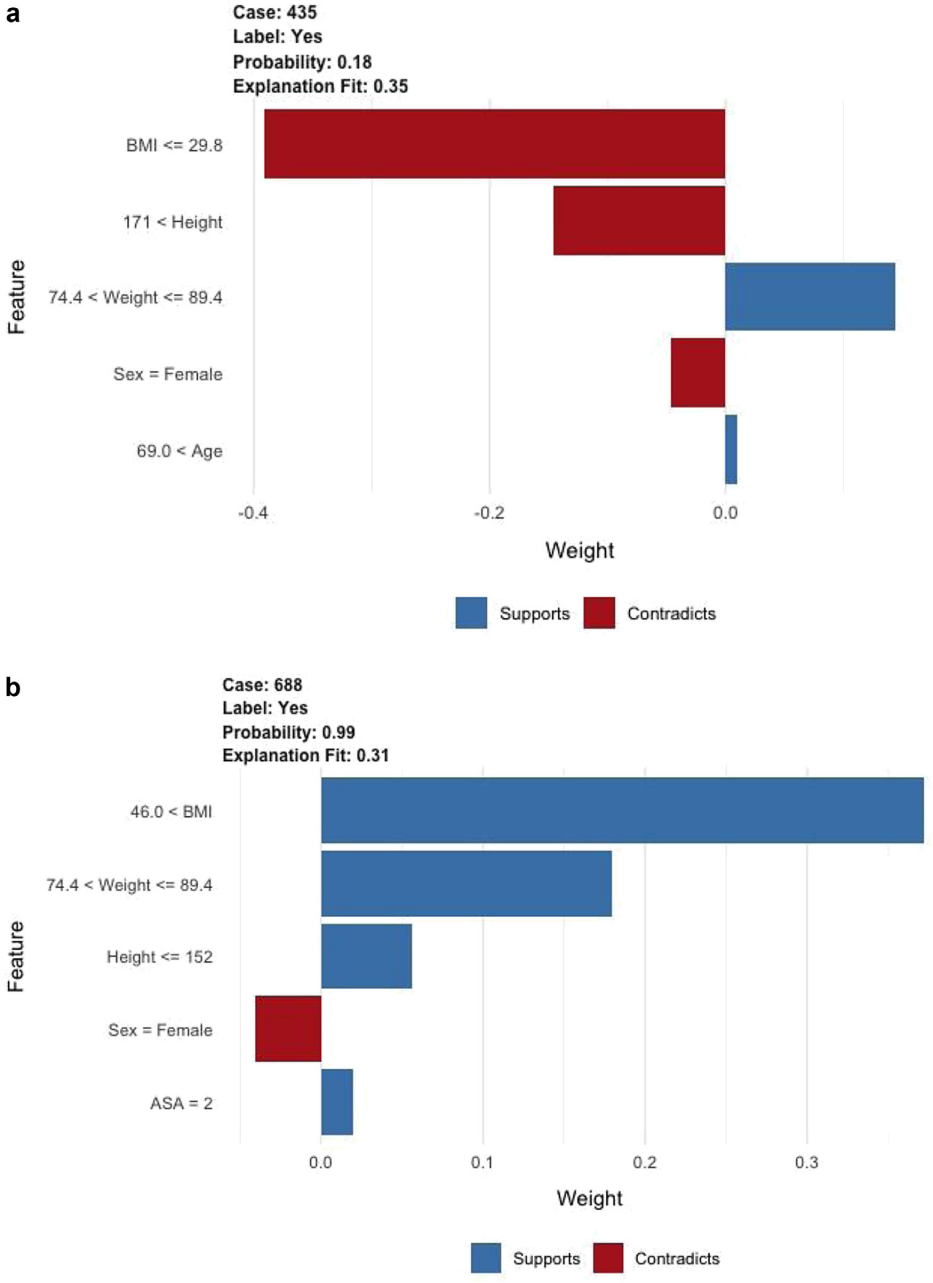
Example of individual patient-level explanations using the stochastic gradient boosting algorithm. Risk profiles for a patient with a predicted femoral component mismatch probability of (a) 18% (class designation: low risk) and (b) 99% (class designation: high risk). The supporting features (blue) represent factors that increased the model prediction of femoral component mismatch, whereas the contradicting features (red) represent factors that decreased the model prediction of femoral component mismatch.

An example of the patient-level explanations and potential clinical utility of the open-source application after external validation is depicted in Figure 5. These 2 cases demonstrate that the model predictions change based on each patient’s unique risk profile and illustrate how model decisions can be easily interpreted and understood using this method.

## Discussion

The main findings of the present study are as follows: 1) Machine learning algorithms predicted those patients at greater risk for mismatch between preoperative templating and final femoral and tibial implant sizes with acceptable to good predictive performance and demonstrated reliability; 2) the predictive accuracy of concordance/mismatch between preoperative templating and final implant sizes using demographic variables was comparable to the accuracy of an experienced arthroplasty surgeon solely using templating; and 3) we created a templating assistance tool that may assist surgeons in selecting their implant sizes by providing them with feedback as it relates to the difficulty of templating on a case-by-case basis. The present findings emphasize the utility of the input parameters chosen for algorithm training and suggest that these parameters may be useful for predicting exact implant sizes. Future studies should focus on using these and other pertinent variables (eg, surgeon preferences for femoral flexion/downsizing, implant bearings) to train machine algorithms for accurate prediction of femoral/tibial TKA implant sizes.

Through iterative learning and algorithm refinement in a large cohort of primary TKA patients, the machine learning models developed in the present study demonstrated reliable accuracy for predicting implant size mismatch. We predicted implant size using 2 different outcomes: 1) a binary outcome in which the prediction was classified as a simple match or mismatch (ie, whether the preoperative templating was likely to exactly match the final implant size or not) and 2) a multiclass outcome in which the algorithm was capable of detecting whether the implant was likely to be undersized, an exact match, or oversized. Although to our knowledge previous literature has not explored the utility of machine learning to predict implant size for TKA, attempts at predictive modeling have been made. Sershon et al. [8] used multivariate linear regression with and without the inclusion of preoperative templating and found that the accuracy of predicting final implant size within the error of one size was as low as 71% for the femoral component and 81% for the tibial component. Although the present study did not attempt to predict exact numeric implant sizes, there are several reasons why machine learning may be better suited for this task than linear regression analyses. First, machine learning is capable of dynamic change and incorporation of new data in a process termed “continual learning.” While linear regression models are static and difficult to modify after initial development, machine learning can adaptively learn the underlying associations of newly presented data while simultaneously retaining previously learned rules. This flexibility is critical because determining the best parameters to use for these models will inform the design of future studies. Second, machine learning algorithms are better equipped to model the complex, nonlinear associations between predictor and outcome variables in big data.

Although developing a cost-effective and reliable means of predicting component size for TKA has been the focus of recent research efforts, an optimal planning tool remains a modern-day unsolved dilemma. The overall prediction accuracy with preoperative templating of an adult reconstruction, fellowship-trained surgeon with over 10 years of experience was 70.1% for the femoral component and 68.5% for the tibial component. In comparison, the accuracies derived from machine learning using demographic variables to detect binary sizing mismatch were 70% for femoral component prediction accuracy and 67% for tibial component prediction accuracy. For the multiclass (ie, undersized vs exact size vs oversized) models, the accuracy of predicted sizing concordance/mismatch improved to 83.9% and 82.9% for femoral and tibial implant size, respectively. Furthermore, these accuracies were derived for prediction of final implant sizes for 7 different implant designs, increasing the generalizability as multiple systems are often used in clinical practice. The diversity in the included implant manufacturer and design also demonstrates the ability of machine learning algorithms to handle heterogeneous pools of data and learn from these data to optimize prediction.

Despite the promise of artificial intelligence in the field of orthopedic surgery, the authors recognize that machine learning will not (and should not) completely replace the training and experience of the adult reconstruction surgeon. Rather, it is more likely that machine learning may serve as a valuable tool to augment decision-making through its capabilities of making predictions at the individual patient level [10]. The open-source application developed in the present study is an example of such a tool. Although this application should not be used in clinical practice until rigorous external validation is performed, it demonstrates the power of machine learning to make individual predictions and may serve as an educational tool. The application is able to take routinely collected information from preoperative evaluations, make real-time predictions in office-based settings, and inform the adult reconstruction surgeon as to whether their template is likely to be mismatched. This tool may “raise a red flag” (or provide reassurance) during templating, especially for residents, fellows, and junior attendings, to potentially take a closer look at templating for particular patients, or to anticipate having more trays in the operating room. The algorithms were capable of achieving this by learning which combinations of these preoperative factors consistently were predictive of implant mismatches vs exact matches during TKA. These learned sets of rules and pattern recognition were then applied and internally validated on an independent testing set of patients to derive the prediction accuracies and performance metrics. This then allowed for determination of quantitative model risk estimate cutoffs that corresponded with scenarios in which preoperative templating were consistently accurate or mismatched with final component sizing (Fig. 4A-D). Currently, the authors are developing machine learning algorithms to predict the numerical component size for femoral and tibial implants used in TKA and recognize that this would also be a valuable decision adjunct; however, the current data set was not amenable to this analysis given the large number of different implant manufacturers and range of sizes.

The accurate prediction of implant sizes used in TKA may allow for consolidation of surgical trays, more efficient operating room processes and streamlined efficiency, and lower hospital costs [4,7]. To this end, the present study is a step in this direction. These algorithms may not only be useful for the adult reconstruction surgeon in preoperative planning but also to implant manufacturers and health-care systems to optimize manufacturing and minimize costs. Future research is warranted to better understand how to integrate machine learning and applications into clinical workflow and subsequently use them in decision-making.

A few study limitations should be considered. Although the current algorithms were developed and internally validated on a large cohort of patients from one large academic and 2 community hospitals, their predictive capabilities may not be entirely generalizable. We do not encourage the use of the open-source application developed in the present study until these algorithms undergo external validation on independent sets of patients from other institutions. However, we do believe that the incorporation of 7 different implant designs contributes to the generalizability of these algorithms and represents a clinically relevant range of implants used in practice. Another limitation inherent in machine learning is the “black box” phenomenon in which the relationships used by the algorithms to inform prediction outputs are not explicitly known. However, through using local agnostic model explanations, we demonstrated how the models arrive at specific predictions and illustrated how each input feature ultimately contributes to the model decision. Furthermore, local agnostic model explanations also contributed to the development and utility of the templating decision tool created in the present study. Finally, the decision as to how to best incorporate such algorithms into clinical workflow is an area of current research and not fully understood. Avenues through which prediction may be augmented include surgeon-specific habits such as downsizing and flexing the component, or variations in cuts based on surgical preference, and the use of radiographic confirmation in cases to assure that implants were not excessively oversized or undersized. Future research in these areas, as well as patient satisfaction and clinical outcomes as a function of the degree of deviation between preoperative planning with templating and the size of the actual implanted components, is warranted.

## Conclusions

ML algorithms predicted templating mismatch with good accuracy. External validation is necessary to confirm the performance and reliability of these algorithms. Predicting sizing mismatch is the first step in using ML to aid in the prediction of final TKA component sizes. Further studies to optimize parameters and predictions for the algorithms are ongoing.

## Conflict of interests

Dr. Levine is a paid consultant for Link Orthopedics, Merete, and Exactech; receives research support from Zimmer and Artelon; receives royalties, financial, or material support from SLACK Inc.; is on the editorial/governing board for SLACK Inc.; and is a board member/committee appointment for AAHKS and AAOS.

For full disclosure statements refer to https://doi.org/10.1016/j.artd.2021.01.006.

## Figures and Tables

**Table S1:** Multiclass classification performance for predicting femoral component sizing mismatch.

Performance metric	Stochastic gradient boosting	Random forest	Support vector machine	Neural network	Elastic-net penalized logistic regression
Accuracy (%)					
Undersized template	74.0	73.7	69.6	60.2	65.9
Correct match	83.9	80.8	80.2	75.8	76.6
Oversized template	70.5	70.4	67.3	66.8	62.1
Sensitivity (%)					
Undersized template	60.3	58.4	51.2	23.7	45.0
Correct match	87.8	83.1	92.9	94.5	87.8
Oversized template	56.5	58.5	45.4	52.3	40.0
Specificity (%)					
Undersized template	87.8	88.9	88.0	96.7	86.8
Correct match	79.9	78.5	67.4	57.1	65.3
Oversized template	84.5	82.4	89.2	81.2	84.1
Multi-class AUC	0.69	0.69	0.66	0.61	0.61

AUC, area under the curve.

**Table S2:** Multiclass classification performance for predicting tibial component sizing mismatch.

Performance metric	Stochastic gradient boosting	Random forest	Support vector machine	Neural network	Elastic-net penalized logistic regression
Accuracy (%)					
Undersized template	59.9	64.2	62.6	63.5	62.4
Correct match	83.8	82.9	83.3	84.5	79.2
Oversized template	58.1	61.7	56.3	53.9	59.3
Sensitivity (%)					
Undersized template	37.9	44.7	49.0	53.4	46.6
Correct match	95.6	96.4	96.4	98.4	90.7
Oversized template	35.4	37.1	24.1	16.9	30.4
Specificity (%)					
Undersized template	81.8	83.7	76.2	73.6	78.1
Correct match	72.0	69.4	70.2	70.2	67.8
Oversized template	80.8	86.2	88.6	91.0	88.2
Multi-class AUC	0.73	0.74	0.73	0.73	0.70

AUC, area under the curve.

**Table S3:** Sub-analysis of model accuracy for predicting femoral/tibial implant sizing mismatch stratified by implant type.

Implant type	Accuracy of model (%)
Femoral component (N = 504)^a^	
Stryker Triathlon	19/22 (86.4)
DePuy Attune	1/1 (100.0)
Biomet Vanguard	11/15 (73.3)
DJ Orthopedics EMPOWR	26/34 (76.5)
Wright Medical Evolution	26/30 (86.7)
Zimmer NexGen	316/364 (86.8)
Zimmer Persona	32/38 (84.2)
Tibial component (N = 504)^a^	
Stryker Triathlon	15/22 (68.2)
DePuy Attune	0/1 (0.0)
Biomet Vanguard	10/15 (66.7)
DJ Orthopedics EMPOWR	22/34 (64.7)
Wright Medical Evolution	25/30 (83.3)
Zimmer NexGen	258/364 (70.9)
Zimmer Persona	22/38 (57.9)

a The sub-analysis was performed on the testing (hold-out) set of data that was not used for model training. The testing set sample size was achieved via data-preprocessing with adaptive synthetic sampling and an 80:20 randomized partition of the study population.

## Appendix A Supplementary data

Conflict of Interest Statement for Levine

Conflict of Interest Statement for Polce

Conflict of Interest Statement for Paul

Conflict of Interest Statement for Kunze
